# 

**Published:** 1900-09

**Authors:** 


					CONTENTS.
Reminiscences of the Bristol Royal
Infirmary.
By Arthur W.
Prichard
103
Abdominal Incisions.
By Thomas
Carwardine, M.S. Lond., F.R.C.S.
204
Spindle-celled Sarcoma of the Hard
Palate.
By W. J. Greer, F.R.C.S.
Irel.
... 211
X-Ray History of a Fracture.
By James Taylor, M.R.C.S.
214
One Thousand Consecutive Indue-
tions of General Anaesthesia.
?y C. Hamilton Whiteford,
M.R.C S., L.R.C.P. ...
217
The,J?ebriates' Acts, 1879?1899.
Wlr.TTAW   H.r A *
By
William Cotton, M.A., M.D.
Edm., D.P.H
The Dietetic Treatment of Zymotic
Enteritis, especially with regard
to the use of Raw Meat Juice.
By F. Percy Elliott, M.B., C.M.
Aberd
225
Progress of the Medical Sciences:
Medicine.
G. Parker, M.D.
229
Surgery.
T. Caryvardine, M.S.
Lond., F.R.C.S.
236
Dermatology.
HenryWaldo,M.D.
240
Reviews of Books
242
Notes on Preparations for the Sick:
The Pasteurisation of Milk.
By
J. Odery Symes, M.D., D.P.H.
275
The Library of the Bristol Medico-
Chirurgical Society 277
Local Medical Notes    282
Scraps
287

				

